# Organophosphorus Pesticides as Modulating Substances of Inflammation through the Cholinergic Pathway

**DOI:** 10.3390/ijms23094523

**Published:** 2022-04-20

**Authors:** Milton Rafael Camacho-Pérez, Carlos Eduardo Covantes-Rosales, Gladys Alejandra Toledo-Ibarra, Ulises Mercado-Salgado, María Dolores Ponce-Regalado, Karina Janice Guadalupe Díaz-Resendiz, Manuel Iván Girón-Pérez

**Affiliations:** 1 Laboratorio Nacional de Investigación para la Inocuidad Alimentaria (LANIIA)-Unidad Nayarit, Universidad Autónoma de Nayarit, Tepic 63173, Mexico; milton.camacho@uan.edu.mx (M.R.C.-P.); carlos.covantes@uan.edu.mx (C.E.C.-R.); gladys_alejandrat@hotmail.com (G.A.T.-I.); ulises.mercado.salgado@gmail.com (U.M.-S.); 2 Centro Universitario de los Altos, Departamento de Ciencias de la Salud, Universidad de Guadalajara, Guadalajara 47610, Mexico; maria.ponce@cualtos.udg.mx

**Keywords:** organophosphorus pesticides, cholinergic system, inflammation

## Abstract

Organophosphorus pesticides (OPs) are widespread insecticides used for pest control in agricultural activities and the control of the vectors of human and animal diseases. However, OPs’ neurotoxic mechanism involves cholinergic components, which, beyond being involved in the transmission of neuronal signals, also influence the activity of cytokines and other pro-inflammatory molecules; thus, acute and chronic exposure to OPs may be related to the development of chronic degenerative pathologies and other inflammatory diseases. The present article reviews and discusses the experimental evidence linking inflammatory process with OP-induced cholinergic dysregulation, emphasizing the molecular mechanisms related to the role of cytokines and cellular alterations in humans and other animal models, and possible therapeutic targets to inhibit inflammation.

## 1. Organophosphorus Pesticides

In recent years, the application of pesticides has increased, as these substances allow pest and disease control in agriculture and livestock, reducing losses in food production, and allowing better control of vectors of human and veterinary diseases [1]. Currently, the most commonly used pesticides worldwide are organophosphorus pesticides [2,3,4], which are insecticides derived from phosphoric or phosphorothioic acid. In 2019, approximately 2 million tons of pesticides were applied globally; in 2020, pesticides reached up to 3.5 million tons, of which approximately one-third consisted of organophosphorus pesticides [5,6].

**Figure 1:**
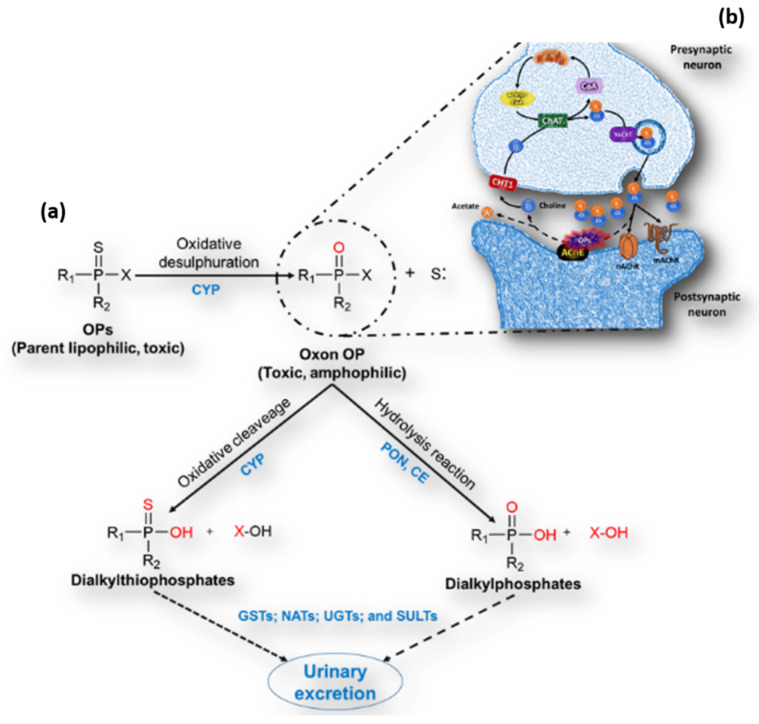
General metabolic pathway of organophosphate pesticides with the neurotoxic mechanism of action. (**a**) The parent organophosphorothionates bioactivated to highly toxic oxon forms by cytochrome P450 through the removal of sulfur attached to phosphorus and insertion of the oxygen atom (oxidative desulphuration) using the reactive and electrophilic iron–oxo intermediate, detoxified by dearylation to form dialkyl thiophosphates (inactive metabolites) or further hydrolyzed to dialkyl phosphates (inactive metabolites) by paraoxonase-1 (PON1) and carboxylesterase (CE) in phase I. Furthermore, phase II involves conjugative reactions carried out by glutathione transferases (GSTs); N-acetyltransferases (NATs); UDP-glucuronosyltransferase (UGTs); and sulphotransferases (SULTs), UDP-glucuronyltransferases (UGT), sulphotransferases (SULT), N-acetyltransferases (NAT), glutathione S-transferases (GST); and is excreted out through urine in a nontoxic form. (**b**) The oxon metabolite phosphorylates the hydroxyl group of the serine present in the active site of the enzyme acetylcholinesterase (AChE) causing ACh accumulation in the nerve synapsis [12].

Organophosphorus pesticides (OPs) are widely used as insecticides, and the use of OPs has replaced organochlorine pesticides, as OPs have limited environmental persistence [1,7]; however, the incorrect handling of these substances during storage, transport, application and the disposal of residues may cause toxic effects on non-target organisms, such as aquatic organisms, domestic and wild fauna, and even humans [8]. Worldwide, more than 3 million acute intoxications and up to 250,000 deaths caused by pesticides are reported annually [9]; OPs reach organisms via inhalation, dermal and oral exposure, the most common being the last one [10]; once inside the organism, these substances are biotransformed (Figure 1a) to highly toxic metabolites (oxon) by the metabolic activation of cytochrome P450 [11], through the elimination of sulfur bound to phosphorus and the insertion of an oxygen atom (oxidative desulfurization). Oxons are detoxified through dearylation and hydrolysis to produce dialkyl phosphates (DAP) or dialkyl thiophosphates, respectively [12], finally by conjugative reactions; these metabolites are excreted out of the body through urine.

### Mechanism of Action of OPs and Toxicity

OPs are designed to inhibit acetylcholinesterase (AChE) activity (Figure 1b) by phosphorylating the hydroxyl group of the serine present in the active site AChE. This interrupts the physiological action of AChE, which degrades the neurotransmitter acetylcholine (ACh), causing its accumulation in the nerve synapses [13], leading to the overstimulation of the muscarinic (mAChR) and nicotinic (nAChR) receptors, and consequently uncontrolled nerve impulses and thus the death of insects [13,14,15,16,17,18,19,20,21]. However, the toxic effects of OPs do not only affect pests; in fact, all organisms that possess cholinergic components can potentially be affected (Table 1) [22,23,24,25,26,27,28,29]. In this sense, humans have a neuronal cholinergic system and—when accidentally or occupationally exposed to these substances—can suffer both acute and chronic effects; the acute effects usually occur minutes or hours after exposure to Ops, and are manifested by clinical signs such as headaches, miosis, diarrhea, muscle weakness, and salivation [30,31], whereas chronic exposure is associated with long-term effects that are complex to attribute to the action of pesticides exclusively. Nevertheless, scientific evidence is growing that these substances induce mutations, epigenetic modifications, tumors, and several types of cancer, as well as cognitive and functional alterations in several physiological systems such as the renal, circulatory, respiratory, endocrine and immune systems [32,33,34,35,36,37]. Currently, several non-neuronal cells, such as pancreatic alpha cells, endothelial cells, placental cells, thrombocytes, and lymphocytes express cholinergic components, which make those cells a target for OPs [38,39,40,41,42,43].

Therefore, several studies have reported that the alteration of the cholinergic system induced by OPs can trigger an inflammatory response and, consequently, pathophysiological alterations [19,44,45,46,47]. Thus, acute OP intoxication has been reported to stimulate an instantaneous and premature robust inflammatory response, whereas chronic exposure to low concentrations of OPs increases inflammatory mediators in a slow but sustained manner [44], or that it could be related to the development of inflammatory diseases such as organophosphate-induced delayed neuropathy (OPIDN) [19], rheumatoid arthritis [45,46], and neuroinflammation [47]. Further studies have shown that exposure to OPs leads to processes of cellular hyperreactivity, synergism with allergens, and the dysregulation of lung physiology, thus promoting susceptibility to asthma development [48,49]. In addition, recent research indicates that exposure to OPs may promote the development of early-stage diabetes mellitus [50].

In the present review, an overview of the experimental evidence linking inflammation to OP-induced cholinergic dysfunction is provided, and the molecular mechanisms through which OPs may induce inflammatory responses are discussed.

## 2. Cholinergic System

**Figure 2:**
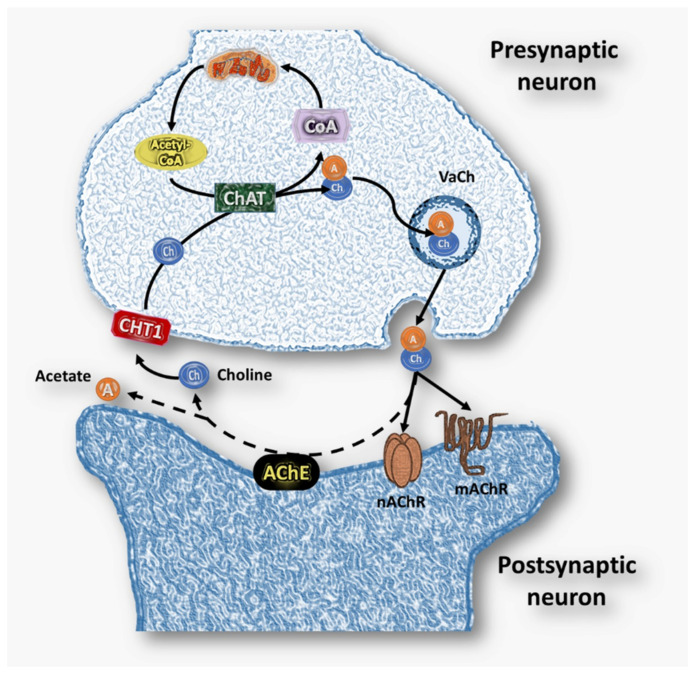
Neuronal cholinergic system. Ch: Choline; A: acetate; ACh: acetylcholine; AChE: acetylcholinesterase; ChAT: choline acetyltransferase; VaCh: ACh vesicles; mAChR: muscarinic ACh receptor; nAChR: nicotinic ACh receptor.

The cholinergic system consists of the biochemical and molecular machinery required to synthesize de novo acetylcholine (ACh), a neurotransmitter that has been conserved throughout evolution [51]. This machinery (Figure 2) consists of synthesis enzymes such as choline acetyltransferase (ChAT, E.C. 2.3.1.6.), storage and transport elements such as ACh vesicles (VaCh), the vesicular ACh transporter (VAChT), the choline transporter (CHT), the muscarinic and nicotinic ACh receptors (mAChR and nAChR, respectively), degradation enzymes such as acetylcholinesterase (AChE, E. C. 3.1.1.7), and non-specific choline esterases such as butyrylcholinesterase (BChE, E.C. 3.1.1.8.) [51]. This set of elements has a fundamental role in the nervous system; however, it is not exclusive to neuronal cells [52,53], as its presence has been demonstrated in other cells such as epithelial (respiratory tract, intestine, skin, urothelium, vagina, placenta, and cornea), endothelial and immune system cells (lymphocytes, macrophages, mast cells, eosinophils, and neutrophils) [54,55], which have been called the “non-neuronal cholinergic system” or “extra-neuronal cholinergic system”; this system is independent of neuronal innervation [39] and has been localized in a wide variety of organisms such as humans and other mammals (rats), as well as lower invertebrates (sponges, corals, ascidians, sea urchins, turbellaria), protozoa, plants, fungi and even bacteria [54].

As previously mentioned, epithelial, endothelial, and immune system cells (macrophages, mast cells, eosinophils, neutrophils, and lymphocytes) are the targets of OPs [43]. In this regard, Kawashima and Fujii [55] documented that mammalian lymphocytes express muscarinic and nicotinic acetylcholine receptors on the cell membrane, and also possess an autonomous cholinergic system, i.e., a non-neuronal cholinergic system through which they produce acetylcholine and degrade it via the enzyme AChE. Other cholinergic components, such as choline acetyltransferase (ChAT), a high-affinity choline transporter, are also expressed in lymphocytes [56]. At present, several studies report the effects of OPs on the vertebrate immune system [1,57,58,59,60,61,62,63,64]; nonetheless, the immunotoxic mechanisms of these substances are not completely elucidated, as OPs could exert a direct cytotoxic effect on lymphocytes, or indirectly, by altering the cholinergic system present in these cells [43,65]. In this way, the lymphocyte cholinergic system could be targeted by OPs in the immunotoxicity phenomenon, which could lead to systemic inflammatory manifestations that manifest as neurological, cardiovascular, and autoimmune diseases [66].

## 3. Immunotoxicity of OPs through the Cholinergic System

In vertebrates, the modulation of the immune response by cholinergic pathways is well established [21,67,68,69]. Processes like the development, proliferation, differentiation and activation of immune cells have been linked to AChRs [70]. The stimulation of mAChRs increases intracellular Ca^2+^ influx, upregulates c-Fos expression, and affects cell proliferation [42]. In addition, mAChRs are related to immune defense, as they modulate inflammatory processes and antibody isotype switching [21,67]. Moreover, antigenic stimuli induce the expression of cholinergic components in leukocytes [71]. Among regulatory processes of inflammation, the cholinergic anti-inflammatory pathway (CAP) plays a prominent role, where the neurotransmitter ACh activates nicotinic receptors of inflammatory cells, with the homopentameric receptor nAChR-a7 being one of the most important participants, leading to a decrease in the synthesis of proinflammatory cytokines and LPS-induced TNF and HMGB1 release [72,73]. Hence, the regulation of cholinergic pathways through specific agonists and antagonists may represent a neuro-immune target in chronic inflammatory diseases.

Immune cell function is highly regulated by classical soluble molecules, such as cytokines, hormones, neurotransmitters, and by cell-to-cell interactions, and it can be directly or indirectly affected by several factors, such as toxic lifestyle, iatrogenic, biotoxic, environmental/occupational, and psychosocial/socioeconomic conditions [74]. However, epidemiological and toxicological evidence suggests that OPs exert immune side effects in both humoral and cell mechanisms (innate or adaptive) [43,75]. Immune response perturbation induced by OPs could be an enhancement (hypersensitivity and autoimmunity) or immunosuppression (susceptibility to infections or neoplastic transformation) [76]. OPs can exert toxic effects through mechanisms unrelated to AChE inhibition, as these compounds can bind to cholinergic receptors [77]. In this regard, both the nerve agents Soman and XV, as well as oxon metabolites of OPs (paraoxon, malaoxon, and diazoxon) can directly interact with cholinergic receptors and modulate the level of receptor expression [78]. The dysregulation of RNA and the protein expression of nAChR (α4 and β2 subunits) after exposure to oxon metabolites was demonstrated in pC12 cells [79]. Likewise, it has been shown that some OPs interact more often with the α4β2 subunits of neuronal nAChRs to inhibit the agonist-induced response [80]. Such a finding suggests that, in addition to AChE inhibition, the inhibition of neuronal nAChRs occurs, thus explaining the massive blocking effect of anti-inflammatory metabolic pathways.

In addition, alterations of the neuronal cholinergic system have been reported in the leukocyte cholinergic system, as it has been reported that exposure to diazinon (DZN) in vivo (3.91 mg/L) causes a decrease in the protein concentration of nAChR and mAChR of the immune cells of Nile tilapia [23]. Similar results were obtained by Charoenying et al. [81], who showed that paraoxon causes cholinergic dysregulation in lymphoma (MOLT-3) and neuroblastoma (SH-SY5Y) cell lines, finding that the lymphocyte extraneuronal cholinergic system has greater susceptibility to OPs than its neuronal counterpart, which could be related to immunotoxicity mechanisms. However, the mechanisms of immunotoxicity induced by OPs such as DZN are not yet fully elucidated, although it has been proposed that the extraneuronal cholinergic system could be related to immunotoxicity [22,81,82].

**Figure 3:**
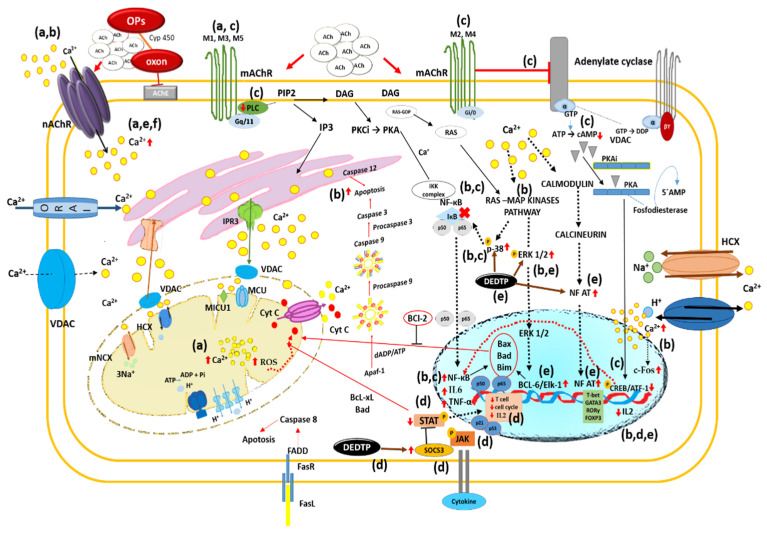
Modulation of signal transduction through the cholinergic system by OP exposure. Exposure to OPs induces AChE inhibition and ACh accumulation, leading to AChR overstimulation. (a) The overactivation of nAChRs and mAChRs leads to increased Ca^2+^ influx, which in turn induces increased ROS in mitochondria. (b) Increased intracellular Ca^2+^ induces activation of p38-MAPK and ERK signaling promoting an inflammatory stage through NF-KB the activation and increased levels of pro-inflammatory cytokines (TNF-alpha and IL-6). In addition, intracellular Ca^2+^ signaling evokes the up-regulation of c-fos expression and IL-2-induced signal transduction in T and B cells, triggering inflammatory responses. (c) OPs cause the inhibition of phospholipase C and decreased CREB phosphorylation and cAMP levels. Reduced CREB phosphorylation promotes NF-κB activation and leads to the degradation of IκB, which allows the release of NF-κB and facilitates its nuclear translocation, where it promotes the transcription of genes involved in pro-inflammatory immune responses. (d) The OP metabolite (DEDTP) promotes the phosphorylation of SOCS3 and the dephosphorylation of STAT5 protein, and leads to the activation of p21, resulting in T-cell arrest. (e) DEDTP also induced the phosphorylation of ERK, JNK, and p38, resulting in the assembly of AP1, ELK,1, and NFAT, which are the major transcription factors involved in the IL-2 autocrine pathway.

Furthermore, it has been reported that the overactivation of nAChRs and mAChRs leads to increased calcium influx [24], which in turn induces increased ROS production, as both processes are highly coordinated in these cell types [83]. Increased intracellular calcium generates mitochondrial stress that promotes ROS production in this organelle (Figure 3a) [84]. Furthermore, the overactivation of cholinergic receptors in leukocytes has a direct impact on cellular functions such as phagocytosis, which was demonstrated using selective acetylcholine receptor agonists and antagonists [21,85].

The onset of OP-induced immune disorders will depend on the type of cells affected. The authors of [86] reported that patients exposed to organophosphates showed the impairment of neutrophil functions (phagocytosis, respiratory burst, adhesion) leading to recurrent infections, in addition to increased risk of upper respiratory tract infections (tonsillitis, pharyngitis, and bronchitis). On the other hand, the functional mutilation of natural killer (NK) cells due to OP intoxication may partly elevate the risk of cancer and viral infections [87,88]. Deeper effects were observed in the immune system, when the activities of antigen-presenting cells (APCs), such as dendritic cells (DCs) and macrophages, were inhibited, given that they play a key role in the elimination of infectious agents and the deployment of cell-mediated immunity [44]. The functional impairment of DCs and macrophages induced by OPs occurs through negative regulation of co-stimulatory molecules (CD80 and CD86), effector molecules (human leukocyte antigen), MHC expression, and phenotypic modulation [89,90,91].

Pesticide-induced immunosuppression is evidently a risk factor for the clinical complication of inflammatory diseases, especially in occupationally or environmentally exposed individuals, which occurs in developing countries. In this context, the current knowledge of molecular mechanisms suggests a direct effect of the exposure to OPs on immunity and inflammatory processes; however, new experiments with epidemiological approaches are key to demonstrating the existing correlation of the exposure to toxic substances, such as OPs, with the degree of susceptibility of organisms to inflammatory diseases.

## 4. Cytokine-Mediated Modulation of the Inflammatory Process by OP Exposure

The consequences of chronic or early-life exposure to pesticides may be extended beyond innate immune dysfunction to the increased risk of late-life chronic inflammatory-based diseases. Immune cells can release a variety of inflammation mediators, activating pro- and anti-inflammatory processes and regulating intracellular pathways [92]. 

It is essential to understand the ways in which OPs affect immune cell activities and, consequently, the function of the immune system, as these substances can induce alterations in the humoral/cellular mechanisms causing a direct impact on lymphoid tissues and immune cell function [93]. While previous studies reported that OPs could affect immunity through different mechanisms [76,94], immune suppression/dysregulation is a major mechanism by which pesticides exert their immunomodulatory activity, affect immunocompetence, and consequently increase the host’s susceptibility to diseases and an array of immune disorders. OPs can affect immunity by interfering with cell signaling pathways, which could result in changes in cytokine production, surface marker expression, and cell activation (Table 2) [76,90,93,95,96,97,98,99,100,101,102,103]. Thus, pro-inflammatory cytokines induce the initiation of inflammation through interaction with Toll-Like Receptors (TLR), IL-1 receptor (IL-1R), IL-6 receptor (IL-6R), and TNF receptor (TNFR). Receptor activation modulates intracellular signaling pathways, including those controlled by the Mitogen-Activated Protein Kinase (MAPK), Nuclear Factor kappa-B (NF-κB), Janus Kinase (JAK)-Signal Transducer, and the Activator of Transcription (STAT). These transcription factors promote cytokine expression, modulating a large number of inflammatory genes, such as IL-1, TNF-α, IL-6, interferons, Transforming Growth Factor (TGF), and chemokines [104].

In this context, it has been shown that exposure to OPs induces the activation of calcium-mediated p38-MAPK and ERK signaling (Figure 3b), promoting an inflammatory stage through NF-KB activation and increased levels of pro-inflammatory cytokines such as TNF-alpha IL-6 [105,106,107]. Lasram et al. [95] reported that malathion induces the release of pro-inflammatory cytokines such as IL-1β, IL-6, and INF-γ. These cytokines are responsible for the activation of nuclear transcription factors such as NF-κB, and are involved in inflammation and the apoptosis of damaged cells. El-Sayed et al. [96] reported that chlorpyrifos can upregulate the expression of some pro-inflammatory markers such as TNF-α and IL-1β, besides the activation of NF-κB, which is an important transcription factor that can be found in the cytoplasm in the form of a dimer of p65 and p50 subunits. Under normal conditions, NF-κB was found to be bound to the inhibitory protein IκB. However, upon exposure to stressful conditions (such as environmental contaminants such as OPs), IκB is phosphorylated, and is separated from the p65 subunit of NF-κB. Consequently, NF-κB is translocated into the nucleus to activate the transcription of pro-inflammatory cytokines like TNF-α and interleukins to instigate inflammatory responses, and consequently to activate the apoptotic pathway (Figure 3b) [96,97], supporting a critical role for NF-κB in transducing diverse environmental stimuli to upregulate cytokine expression in inflammatory cells.

Moreover, it has been shown that exposure to OPs causes phospholipase C inhibition, as well as decreased CREB phosphorylation and decreased levels of cAMP (Figure 3c) and mAChR mRNA (M1, M2, M3) [108,109]. CREB phosphorylation is a focal point for multiple signaling cascades, and is recognized to play a critical role in neuronal development, synaptic plasticity, memory function, regeneration, and cell survival in response to diverse types of stress [110]. The reduced phosphorylation of CREB by exposure to OPs may contribute to neurobehavioral deficits, and may also affect the transcription of genes associated with learning, memory, and synaptic plasticity. It has recently been suggested that the persistence of long-term memories may depend on the activation of the cAMP/MAPK/CREB transcriptional pathway in the hippocampus [111,112]. CREB also plays many different roles in immune function by promoting anti-inflammatory immune responses, such as the inhibition of NF-κB activity, the induction of IL-10, and the generation of T-regs [113]. However, reduced CREB phosphorylation induced by OP exposure promotes NF-κB activation causing a cascade of signaling events that ultimately lead to the degradation of IκB (Figure 3c), which allows NF-κB release and facilitates NF-κB nuclear translocation, where it promotes the transcription of genes involved in pro-inflammatory immune responses [113,114,115].

Macrophages also play an important role in OP-induced inflammation [103]. Ogasawara et al. [102] showed that OPs not only enhance the production of pro-inflammatory markers such as IL-6 and TNF-α but also the number of macrophages, and increase the expression of cyclooxygenase (COX)-2 and inducible nitric oxide synthase enzymes as a major source of ROS. In this way, oxidative stress can stimulate the expression of inflammatory transcription factors, which are crucial regulatory components in the induction of inflammatory responses [96].

Nevertheless, in the cells, there are a plethora of negative regulators of inflammatory signaling pathways that operate in a negative feedback fashion (i.e., those pathways which are inducible by inflammatory signals). These include the suppressor of cytokine signaling (SOCS) proteins, negative regulators of Janus kinase–signal transducer and activator of transcription (JAK-STAT) signaling, and A20, a negative regulator of nuclear factor-kB (NF-kB) signaling [116]. However, this plethora of negative regulators of inflammatory signaling pathways can also be modulated by OP exposure [117,118,119,120,121]. Esquivel-Sentíes et al. [117] proposed that the alteration of the function and components of the immune system may be related to the sequence and intensity of the phosphorylation and dephosphorylation of protein kinases, an essential mechanism that controls the function of the immune system. SOCS3 (suppressor of cytokine signaling 3) is a critical molecule in this process, as it functions as a negative regulator of cytokine signaling. SOCS3 regulates STAT by inhibiting the phosphorylation of STAT5 affecting cell proliferation [118,119].

Recent reports have shown that metabolites (dialkyl phosphates) generated by the biotransformation of OPs as diethyl thiophosphate (DETP) and diethyl dithiophosphate (DEDTP) modify the phosphorylation status of STAT5 (Figure 3d) proteins, and thus produce several immunomodulatory effects, for instance, the reduction of CD25 and CD4 expression, the reduced secretion of IL2, and the altered signalization of IL-2R [117,120]. Esquivel-Sentíes et al. [117] reported that DEDTP treatment in human T lymphocytes increases SOCS3 phosphorylation and decreases STAT5 phosphorylation, resulting in the arrest of T cell proliferation (Figure 3d). On the other hand, Lima et al. [121] reported that DEDTP can trigger SOCS3-mediated cell cycle arrest that initiates a feedback mechanism associated with the expression of p21 and p53. DEDTP also induced the phosphorylation of ERK, JNK, and p38 [117], which results in the assembly of AP1, ELK,1, and NFAT, which are the main transcription factors involved in the autocrine IL-2 pathway (Figure 3e) [117,121,122].

Regarding the cholinergic system, acute OP poisoning induces the overstimulation of cholinergic receptors due to the accumulation of ACh at immunological synapse, evoking intracellular Ca^2+^ signaling, the upregulation of c-fos expression (Figure 3b), and IL-2-induced signal transduction in T cells and B cells, as well as triggering inflammatory responses in macrophages [44,55,76]. In contrast, chronic OP poisoning through the down-regulation of cholinergic receptors may trigger cholinergic anti-inflammatory pathways, which result in the suppression of T-cell activity, predisposition to cancer, and certain infections [44,76,82,83].

## 5. Therapeutic Strategies to Mitigate the Long-Term Inflammatory Effects of Acute OP Intoxication

The canonical mechanism of the neurotoxicity of OPs is AChE inhibition [123]; thus, acute AChE inhibition (>60 to 80%) can induce a clinical condition termed cholinergic crisis [47], which is characterized by peripheral parasympathetic symptoms, the depression of central breathing control, seizures that can quickly progress to status epilepticus (SE), and the death of the intoxicated individual [124,125]. The conventional treatment to control OP-induced cholinergic seizures is based on the use of drugs such as atropine (a peripheral muscarinic receptor antagonist) [47], pralidoxime (a reactivator of AChE activity) [126] and benzodiazepine (which reduces seizure activity) [124,127]; however, in severe cases of OP poisoning, these agents are not effective. Furthermore, OP intoxication can result in long-term alterations, which are manifested by cognitive dysfunction, affective disorders, or spontaneous recurrent seizures (SRS) [30,128,129,130,131,132], which are linked to neuroinflammatory processes [128].

In the neuroinflammatory disorder induced by OPs, microglia cells play a central role in regulating the production of pro-inflammatory cytokines that eventually damage neurons and exacerbate the course of neurodegenerative alterations [133]. Therefore, new pharmacological therapies should focus urgently on the inactivation of microglia and the inhibition of the inflammatory response. In this regard, it has been shown that blocking intracellular Ca^2+^ release, inhibiting NLRP3-inflammasome (NF-κB and MAPK blockers), and controlling ROS production (NADPH oxidase inhibitors -Nox1, Nox2, and Nox4) may be important therapeutic targets to counteract the neuronal damage caused by OPs [47,66,134,135,136,137,138,139].

## 6. Lower Vertebrates as a Biomedical Model

Lower vertebrates have become relevant in the field of biomedical research, given that such vertebrates offer advantages over different study models (e.g., mice). An example of these are fish, which belong to the phylogenetically oldest group of vertebrates, including more than half of the vertebrates on the planet; the vast majority of fishes are teleosts (teleosts, possessing a bony skeleton), and some are highlighted for both their ecological and economic significance, while other species are widely used as biological models for genomic studies and developmental biology [65,140]. Furthermore, as these organisms are the first to present adaptive immune mechanisms, the study of the immune system in these organisms is of great relevance, as it provides information on the evolution of the immune system in vertebrates, thus supporting the knowledge of basic aspects of immunology, and thus the possible treatment of emerging diseases in humans and other animals. Wilson [141] proposed that teleost fishes can be a good model for translational research because they possess mechanisms of innate and adaptive immunity (TLR toll-like receptors, cytokines, complement molecules, B cells, T cells, and immunological memory) which are very similar to those of higher mammals.

Furthermore, teleost fish have also been used as bioindicators of pollution, as they can respond to environmental pollution through alterations in physiology or through the storage of pollutants [142,143]. The use of fish as bioindicators is of great importance for several reasons, due to their sensitivity to environmental stressors, wide geographic distribution, presence in the food chain, and ease of adaptation to captivity, which permits the evaluation of the effect of environmental stressors under controlled conditions [144]. Given this background, our research group has used Nile tilapia (*Oreochromis niloticus*) and guppy fish (*Poecilia reticulata*) as bioindicator organisms and biomedical study models, to elucidate the mechanism of immunotoxicity by OPs (Table 3).

Initial studies demonstrated that OPs (chlorpyrifos and diazinon) cause immunotoxic effects by altering the physiological parameters of leukocytes, such as decreased phagocytic capacity [145,146,147], increased respiratory burst [61], and the dysregulation of IgM concentration and lysozyme activity [61,148], in addition to oxidative damage in liver and gill proteins [149]. Subsequently—derived from Kawashima and Fujii [55], who reported that mammalian lymphocytes possessed all of the biochemical and molecular machinery necessary to synthesize ACh de novo—we were prompted to search for this cholinergic system in the mononuclear cells of Nile tilapia, demonstrating not only the presence of the extraneuronal cholinergic system in these cells but also that when the organisms were exposed to DZN, the activity of AChE was inhibited and the concentration of ACh increased [23], suggesting that the lymphocyte cholinergic system could be targeted by OPs in the immunotoxicity phenomenon [43]. Later, in order to elucidate a possible mechanism of immunotoxicity by OPS, the effect of DZN and its metabolite oxon (diazoxon) on intracellular Ca^2+^ flux and pERK1/2, parameters that play a fundamental role in cell signaling were assessed, in addition to mitochondrial membrane potential (ΔΨm), ROS, NETs, senescence, and apoptosis, which were determined in Nile tilapia leukocytes, demonstrating that DZN and its metabolite oxon alter intracellular Ca^2+^ and pERK1/2 signaling, leading to the depolarization of the mitochondrial membrane by increased ROS, leading cells to NETosis, senescence and/or death by apoptosis [107,147,150,151]. Likewise, it was demonstrated that tilapia leukocytes express mAChR (M2, M3, M4, M5A) [152], and that after exposure to diazoxon, the expression of muscarinic receptors (M3, M4, M5) and nAChR-β2 decreases [152].

On the other hand, guppy fish (*P. reticulata*) have also been used by our research group as a model organism to study the toxic effects of these substances used by the Mexican Ministry of Health to control vectors that transmit viral diseases such as dengue, chikungunya, and Zika. The results of these investigations indicate that exposure (7 and 21 days) in vivo to temephos (0.5 mg/L), an OP, causes cholinergic alterations (the inhibition of AChE and the accumulation of the neurotransmitter ACh) in muscle tissue [153]. In addition, it causes a decrease in phagocytic capacity [154] and a decrease in leukocyte viability, inducing apoptosis and necrosis. The data even reveal that temephos induces apoptosis up to 35 days post-exposure, indicating recovery up to 70 days [155].

At present, our research group is working on the effect of diazinon and its metabolite oxon on key molecules involved in cell signaling, aiming to elucidate a possible mechanism of immunotoxicity by these substances. In this sense, we are focusing on the effects of OPs on the expression of cytokines (anti-inflammatory and pro-inflammatory) and master transcription factors (T-bet, GATA-3, RORγt, and FOXP3), as well as on the phosphorylation of JAK/STAT, and levels of cAMP, DAG, and IP3.

**Table 3:** Effect of OPs on the molecular and cellular parameters of fishes leukocytes as a study model.

OPs	Dose	Exposure Time	Effects	Tissue/Cell Line	Organism Model	References
Diazinon	LC_50_-7.830 ppm, ½ LC_50_-3.915 ppm	96 h	↓ AChE activity ↑ ACh levels	Spleen mononuclear cells	Nile tilapia (*O. niloticus*)	[22].
Diazinon	0.97, 1.95 and 3.91 mg/L	6, 12 and 24 h	↓ AChE activity ↓mAChR, nAChR concentration and ↑ ACh levels.	Spleen mononuclear cells	Nile tilapia (*O. niloticus*)	[23].
Diazoxon	1 nm, 1 µM, and 10 µM	24 h	↓ (M3, M4, M5) receptors and nAChR-β2 expression.	Spleen mononuclear cells	Nile tilapia (*O. niloticus*)	[24].
Diazinon	1.96 mg/L	96 h	↑ Respiratory burst and IgM concentration	Spleen mononuclear cells	Nile tilapia (*O. niloticus*)	[61].
Diazinon	0.97, 1.95 and 3.91 mg/L	6 and 24 h	Alterations in Ca^2+^ flux and pERK 1/2. ↑ Cellular senescence ↓ mitchondrial membrane. potential ↑ apoptotic cells.	Spleen mononuclear cells	Nile tilapia (*O. niloticus*)	[107].
Chlorpyrifos	0.422 and 0.211 mg/L)	96 h	↓ Phagocytic Capacity.	Peripheral blood	Nile tilapia (*O. niloticus*)	[145].
Diazinon	LC_50_-7.830 ppm	96 h	↓ Phagocytic capacity and cellular proliferation.	Spleen mononuclear cells	Nile tilapia (*O. niloticus*)	[146].
Diazinon	0.97, 1.95 and 3.91 mg/L	6 and 24 h	↑ Reactive oxygen species ↓ Phagocytic activity	Peripheral blood mononuclear cells	Nile tilapia (*O. niloticus*)	[147].
Chlorpyrifos	0.051 mg/L	96 h	↓ IgM levels and deregulation in lysozyme activity.	Spleen mononuclear cells	Nile tilapia (*O. niloticus*)	[148].
Diazinon	0.97, 1.95 and 3.91 mg/L	12 and 24 h	↑ Protein oxidative damage.	Liver and gills	Nile tilapia (*O. niloticus*)	[149].
Diazinon	0.97, 1.95 and 3.91 mg/L	6 and 24 h	↑ Neutrophil extracellular traps (NETs) induction.	Spleen mononuclear cells	Nile tilapia (*O. niloticus*)	[150].
Diazoxon	1 nm, 1 µM, and 10 µM	1 h and 2 h	↓ Ca^2+^ flux against PMA and ionomycin stimulation. ↓ ERK1/2 phosphorylation. ↓ Mitochondrial membrane potential. ↑Apoptotic and cellular senescence.	Spleen mononuclear cells	Nile tilapia (*O. niloticus*)	[151].
Temephos	10 mg/L	7 and 21 days	AChE inhibition ↑ ACh levels	Smooth muscle	*Guppy fish (Poecilia reticulata)*	[153].
Temephos	10 mg/L	7 days	↓ Phagocytic capacity	Spleen mononuclear cells	*Guppy fish (P. reticulata)*	[154].
Temephos	10 mg/L	7, 14, and 21 days	↑ Leucocytes death	Spleen mononuclear cells	*Guppy fish (P. reticulata)*	[155].

↑ increase ↓ decrease; ACh: acetylcholine; AChE: acetylcholinesterase; mAChR: muscarinic acetylcholine receptor; nAChR: nicotinic acetylcholine receptor; ROS: reactive oxygen species; NETs: neutrophil extracellular traps. IgM: immunoglobulin M; ERK: extracellular signal-regulated kinase.

## 7. Conclusions

In conclusion, the present review clearly shows that OPs are substances that, despite being designed for insect control, affect the physiology of non-target organisms, including humans. Due to the mechanism of action of OPs, these substances alter the activity of the cholinergic system, which significantly influences the transcription, synthesis, and release of inflammatory mediators such as cytokines. Consequently, acute and chronic exposure to OPs may be related to the development of chronic degenerative pathologies, as well as allergies or immunosuppression phenomena, alterations in which inflammatory components play a central role.

## Figures and Tables

**Table 1:** Effects on cholinergic systems by OP exposure.

OPs	Dose	Exposure Time	Cholinergic Effects	Tissue/Cell Line	Organism Model	References
Diazinon	LC_50_-7.830 ppm, ½ LC_50_-3.915 ppm	96 h	↓ AChE activity ↑ ACh levels	Spleen mononuclear cells	Nile tilapia *(Oreochromis niloticus)*	[22].
Diazinon	0.97, 1.95 and 3.91 mg/L	6, 12, and 24 h	↓ AChE activity ↓ mAChR, nAChR concentration and ↑ ACh levels.	Spleen mononuclear cells	Nile tilapia (*O. niloticus*)	[23].
Diazoxon	1 nm, 1 µM, and 10 µM	24 h	↓ (M3, M4, M5) receptors and nAChR β2 expression.	Spleen mononuclear cells	Nile tilapia (*O. niloticus*)	[24].
Paraoxon	1 mg/kg	6 and 24 h	↓ mAChR M2 function ↑ ACh levels. ↑ mAChR M3 stimulation	Peripheral blood	Guinea Pig	[25].
Chlorpyrifos	LD_50_ ^1^/_3_ LD_50_	48 h	↓ ChAT activity ↓ AChE activity	Cerebral cortex	Male Rat	[26].
Chlorpyrifos	1 mg/Kg	1 h and 6 h	↓ ChAT activity, nAChR α4, and α7 expression ↓ VAChT expression	Forebrain Peripheral blood	Human apoE-TR mice	[27].
Monocrotophos	0.01, 0.10, or 1.00 mg/L	N/A	↓ ChAT activity ↓ AChE activity	Embryos	Sea urchin *(Hemicentrotus pulcherrimus)*	[28].
OPs	Acute exposure	N/A	↓ BuChE activity	Peripheral blood	Human	[29].

↑ increase ↓ decrease; ACh: acetylcholine; AChE: acetylcholinesterase; mAChR: muscarinic ACh receptor; nAChR: nicotinic ACh receptor; ChAT: choline acetyltransferase; VAChT: vesicular ACh transporter; BuChE: butyrylcholinesterase.

**Table 2:** Reports of the modulation of the inflammatory process mediated by cytokines due to exposure to OPs.

OPs	Dose	Exposure Time	Effects of Cytokines	Inflammation Results	Organism Model	References
Chlorpyrifos, dimethoate	0–1000 μM	24 h	IL-10 was significantly downregulated	↓ DC-specific cell surface markers (i.e., CD83 and CD209). Inhibition of Akt family	DC, differentiated from the monocyte cell line THP-1	[90].
Chlorpyrifos	0, 001, 10 μM	24 h	↓ Expression of IL-1β and TNF-α	Biphasic responses of lysosomal enzyme activity. inhibition NO release	Macrophages from mouse peritoneum	[93].
Malathion	200 mg/kg b.w./day	28 days	↑ Expression of IL-1β, IL-6 and IFN-γ	↑ Activities of hepatocellular enzymes in plasma, lipid peroxidation index, CD3+/CD4+ and CD3+/CD4+ percent	Adult male Wistar rats	[95].
chlorpyrifos	3.375–13.5 mg/kg	28 days	↑ Expression of IL-1β and TNF-α	↑ Activation of NF-kB, cleaved caspase 3 and HO-1 and Nrf-2 pathway Cellular damage in organs	Male Wistar rats	[96].
Parathion, chlorpyrifos, and diazinon	1–100 μM	24 h	↑ Expression of TNF-α, IL-1β PDGF (platelet-derived growth factor) and TGF-β (transforming growth factor-β). the of TNF-α protein.	↑ NF-κB activation and ↓AChE activity	THP1 cells differentiated into macrophages	[97].
Chlorpyrifos	6.75 mg/kg	8 weeks	↑ Expression of IL-6, TLR-2, IL-1β, TNF-α, and NLPR3	↑ Expression of apoptotic genes (*Caspase* 3, *Caspase* 9, *Caspase* 8 *and Bax*)	Male rats	[98].
Triphenyl phosphate	0, 50, or 150 mg/kg	30 days	↑ Expression of IL-6 and TNF-α	↑ Inflammation in the thalamus and hippocampus. MAPK signaling pathways were significantly affected.	Male mice (C57/BL6)	[99].
Malathion	27 mg/kg (1/50 of LD_50_)	30 days	↑ Expression of IF-γ, IL1-β, TNF-α, and NFĸB	↓AChE levels in serum (30%) and liver (25%) compared to the control group. Lipid peroxidation.	Rats	[100].
Chlorpyrifos	0.3–300 μM	24 h	↑ Expression of IL-1β and NLRP3	↑ Oxidative stress production (NO, MDA, and O_2_∙)	BV-2 microglial cells.	[101].
Diazinon	10–100 μM	24 h	Induce expression of TNF-α and IL-6	↑ ROS generation. Induced expressions of COX-2, iNOS, and cell-surface molecules CD40, CD86, and MHC class II. ↓phagocytic activity	RAW264.7 cells	[102].
Parathion, Malathion, paraoxon and malaoxon	100–2000 µmol/L	24 h	↑ Expression of IL-6, GM-CSF and MIP-1α	↓Viability, intracellular GSH and phosphorylation of STAT3. ↑Phosphorylated p38MAPK	Rat precision-cut lung slices	[103].

↑ increase ↓ decrease; IL-6: Interleukin 6; IL-2: Interleukin 2; IL-1β: Interleukin 1 beta; TLR-2: Toll-like receptor; TNF- α: Tumor necrosis factor alpha; NLPR3: NLR family pyrin domain containing 3; IFN-γ: Interferon gamma; GM-CSF: Granulocyte-macrophage colony-stimulating factor; MIP-1α: Macrophage inflammatory protein; IL-10: Interleukin 10.

## Data Availability

Not applicable.
